# Platelets in Wound Healing: What Happens in Space?

**DOI:** 10.3389/fbioe.2021.716184

**Published:** 2021-10-25

**Authors:** Laura Locatelli, Alessandra Colciago, Sara Castiglioni, Jeanette A. Maier

**Affiliations:** ^1^ Department of Biomedical and Clinical Sciences L. Sacco, Università di Milano, Milan, Italy; ^2^ Department of Pharmacological and Biomolecular Sciences, Università di Milano, Milan, Italy; ^3^ Interdisciplinary Centre for Nanostructured Materials and Interfaces (CIMaINa), Università di Milano, Milan, Italy

**Keywords:** platelets, microgravity, platelet rich plasma, wound healing, regeneration

## Abstract

Beyond their fundamental role in hemostasis, platelets importantly contribute to other processes aimed at maintaining homeostasis. Indeed, platelets are a natural source of growth factors and also release many other substances—such as fibronectin, vitronectin, sphingosine 1-phosphate—that are important in maintaining healthy tissues, and ensuring regeneration and repair. Despite rare thrombotic events have been documented in astronauts, some *in vivo* and *in vitro* studies demonstrate that microgravity affects platelet’s number and function, thus increasing the risk of hemorrhages and contributing to retard wound healing. Here we provide an overview about events linking platelets to the impairment of wound healing in space, also considering, besides weightlessness, exposure to radiation and psychological stress. In the end we discuss the possibility of utilizing platelet rich plasma as a tool to treat skin injuries eventually occurring during space missions.

## Introduction

In the adult, approximately one trillion platelets circulate in the blood. These disc-shaped anucleate cells arise from the fragmentation of the cytoplasm of megakaryocytes, residing in the bone marrow. Upon release in the bloodstream, platelets circulate for approximately 10 days before being cleared by the splenic and hepatic reticuloendothelial system (Dowling et al., 2010)*.* In specific conditions, they undergo apoptosis through the intrinsic pathway (McArthur et al., 2018) and activate autophagy, which importantly modulates their function (Banerjee et al., 2019). Platelets contain mitochondria, display a range of coding and non-coding RNAs, synthesize some proteins and store a considerable number of preformed bioactive molecules in uniformly distributed secretory granules derived from megakaryocytes (Gianazza et al., 2020). Beyond the lysosomes containing a panoply of hydrolases, the most abundant are the *α* granules (Blair and Flaumenhaft, 2009), which account for about 10% of the platelet volume and mostly bud from the trans-Golgi network. Alpha granules contain hundreds of proteins such as coagulation factors, growth factors (GFs), adhesive molecules, pro- and anti-angiogenic factors, cytokines and chemokines (Chen et al., 2018). After their release, these proteins potentiate platelet responses in an autocrine fashion or target other cell types through paracrine mechanisms. It is now clear that the content of *α* granules is heterogeneous and this might determine a differential exocytosis of granule’s cargo in response to distinct stimuli. Dense granules, usually 3–8 per platelet, originate from exosomes and contain small molecules among which serotonin, histamine, calcium (Ca^2+^), magnesium, ADP, ATP, pyrophosphates, and polyphosphates, all important to magnify platelet activation (Ambrosio and Di Pietro, 2017)*.* T granules were the last to be identified as an electron-dense tubular system-related compartment containing toll-like receptor (TLR) 9 and protein disulphide isomerase (Thon et al., 2012). Platelet granule release is essential for the full repertoire of platelet activities.

## Platelets

Along with their traditional role in hemostasis, increasing experimental and clinical evidence points to platelets as relevant modulators of some physio-pathological processes such as inflammation, immunity, wound healing and tissue regeneration, because they release GFs, cytokines, and extracellular matrix modulators that sequentially promote angiogenesis, restoration of damaged connective tissue, proliferation and differentiation of mesenchymal stem cells into tissue-specific cell types (Doucet et al., 2005).

### Platelets and Hemostasis

Platelets are sentinels of vascular integrity as they sense and respond to perturbations in the blood and disruption of the endothelial layer (Becker et al., 2018). Physiologically, platelets flow in close proximity to the endothelial cells (Becker et al., 2018), which continuously release anti-platelets molecules, such as nitric oxide and Prostaglandin I2 (PGI2), or prostacyclin. Moreover, the normal healthy endothelium is covered by the glycocalyx, a 0.5–5 µm-thick (Uchimido et al., 2019) proteoglycan rich structure that prevents endothelial-platelet interaction. After a damage to a blood vessel, platelets adhere to the vascular wall through the engagement of different receptors which bind to cellular and extracellular matrix constituents of the vessel wall. Initially, the platelet receptor glycoprotein (GP) Ib-IX-V complex tethers immobilized von Willebrand factor (vWF), a multimeric adhesive protein secreted from activated endothelial cells. This interaction allows the binding of platelet collagen receptor GpVI to its ligand in the uncovered subendothelial matrix and triggers intracellular signals that activate integrins, which are involved in cell-cell and cell-matrix interactions, thereby leading to platelet stable adhesion, activation and aggregation (Varga-Szabo et al., 2008). The adherence of platelets to the vascular wall activates them to undergo a dramatic shape change and to release the content of their granules and also extracellular vesicles (Estevez and Du, 2017; Lopez et al., 2019). These events result from cytoskeletal rearrangements through the rapid reorganization of microtubules and the polymerization of actin, which is prompted by increased intracellular Ca^2+^ initially due to its release from intracellular stores and then to its entry through the plasma membrane (Varga-Szabo et al., 2009). The cytoskeleton, then, directs the centralization of the granules, followed by their fusion mainly with the open canalicular system (OCS), a tunneling network that markedly increases platelet surface area, but also with the plasma membrane (Heijnen and van der Sluijs, 2015). This release reaction is very complex and tightly controlled by intracellular kinases, proteases and Ca^2+^. To further complicate the scenario, it is reported that, in parallel with the degranulation, also microparticles and exosomes, both implicated in intercellular communication, are released and, again, Ca^2+^ plays a prominent role (Melki et al., 2017). Within minutes, platelets aggregate to form the primary hemostatic plug. The secreted microparticles, together with the transbilayer movement of negatively charged phospholipids, provide binding sites for components of the coagulation system with the efficient production of thrombin, which cleaves fibrinogen in fibrin. Fibrin recruits more platelets, and also erythrocytes and leukocytes, and consolidates the initial plug into the so called secondary hemostatic plug. Concomitantly, counter-regulatory pathways are activated to limit the extension and the dimension of the plug (Palta et al., 2014). Platelet adhesion, activation and aggregation are finely tuned by a plethora of pre-formed and neo-synthesized mediators (Nieswandt et al., 2011).

While all the aforementioned events are fundamental to plug holes in injured vessels thus protecting against blood loss, an uncontrolled and dysregulated activation of hemostasis leads to the formation of an intravascular clot which partially or completely blocks the lumen of the vessel, with relevant clinical implications (Satoh et al., 2019). This process is known as thrombosis and is triggered by the so-called Virchow’s triad featured by endothelial damage, altered blood flow and hypercoagulability. On the contrary, impaired hemostasis leads to bleeding diathesis because primary hemostasis is impaired (Vinholt, 2019).

### Beyond Hemostasis: Platelets, Vascular Integrity and Inflammation

Platelets function as gatekeepers of the integrity of the vascular wall through a complex bidirectional communication with the endothelium. In the bone marrow, megakaryocytes constitutively secrete Vascular Endothelial Growth Factor (VEGF) and other angiogenic factors, which promote the survival of bone resident microvascular endothelial cells by upregulating the antiapoptotic protein Bcl2. Conversely, bone marrow endothelial cells support the proliferation and differentiation of the megakaryoblasts as well as megakaryocytic fragmentation by releasing specific trophic cytokines (Nachman and Rafii, 2008). Under physiological conditions, a very low grade platelet activation is likely to occur as a response to rheologic events, thus accounting for a tonic, controlled release of low amounts of endothelial trophogens-VEGF, angiopoietin 1, Brain-Derived Neurotrophic Factor (BDNF), Sphingosine-1-Phosphate (S1P)- stored in the granules or for their exposure on the cell surface (Randriamboavonjy and Fleming, 2018). VEGF and BDNF function as survival factors for the endothelium of mature vessels (Donovan et al., 2000), whereas angiopoietin 1 and S1P contribute to the stabilization of the blood vessels (Gavard et al., 2008; Xiong and Hla, 2014). Consequently, the structural and functional integrity of the vascular-endothelium cadherin complex is preserved, thus maintaining the stability of the vessels. Accordingly, thrombocytopenia is associated with alterations of the intercellular adherens junctions because of the disassembly of cadherin complexes, which culminates with the extravasation of erythrocytes into the neighboring tissues (Nachman and Rafii, 2008).

Platelets also participate in inflammation. They express toll-like receptors, which initiate the innate immune response, and bind components of the complement system, thus resulting in the formation of the membrane attack complex that lyses pathogens (Li et al., 2017; Eisinger et al., 2018). Moreover, platelets accumulate microbicidal proteins, i.e., thrombocidin 1 and 2, in their granules (Eisinger et al., 2018). Consequently, it is not surprising that thrombocytopenia is considered as an independent risk factor of mortality in sepsis (Assinger et al., 2019).

While in physiological conditions platelets dampen neutrophil degranulation and histotoxic functions, in an inflammatory environment they directly interact not only with the activated endothelial cells, but also with leukocytes. Furthermore, platelets release chemokines that recruit leukocytes and stimulates their adhesion to the endothelium by upregulating endothelial adhesion molecules which grant firm adhesion and extravasation (Assinger et al., 2019). Neutrophils are central in innate immunity and are rapidly engaged at the site of inflammation. Platelets enhance neutrophil’s phagocytosis and production of free radicals, and induce the formation of Neutrophil Extracellular Traps (NETs) that protect against pathogens but can also occlude the vasculature or cause immune dysfunction (Lisman, 2018). In parallel, neutrophils secrete proteases that amplify platelet responses by activating protease-activator receptors (Lisman, 2018).

Activated platelets also release C-C Chemokine Ligand 5 (CCL5), which recruits monocyte and T-lymphocytes and preludes their transmigration towards the site of inflammation (Randriamboavonjy and Fleming, 2018). In particular, platelets regulate T-lymphocyte trafficking and activation (Randriamboavonjy and Fleming, 2018). On the other hand, T cells trigger platelet to release CCL5, which further engages T cells (Margraf and Zarbock, 2019).

It should be recalled that, while orchestrating host defense, platelets continue to serve as gatekeepers of the vascular wall to secure vascular integrity (Ho-Tin-Noé et al., 2018). Moreover, it is emerging that platelets also secrete pro-resolving mediators of the lipoxin family, which grant the transition from the inflammatory to the resolution phase (Rossaint et al., 2018).

### Platelets and Wound Healing

**FIGURE 1:**
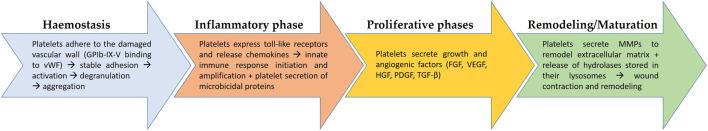
The role of platelets in the different sequential steps of wound healing. In wound healing, four steps are recognized, i.e., hemostasis, inflammation, proliferation, and remodeling/maturation. From the beginning of the process platelets play a fundamental role, starting from the aggregation and formation of a clot to stop bleeding (hemostasis). They also contribute to the recruitment of immune cells (inflammatory phase), to the formation of granulation tissue (proliferative phase), to the remodeling and contraction of the wound to reconstitute structural continuity and, possibly, function. vWF, von Willebrand factor; FGF, Fibroblast Growth Factor; VEGF, Vascular Endothelial Growth Factor; HGF, Hepatocyte Growth Factor; PDGF, Platelet-Derived Growth Factor; TGF-β, Transforming Growth Factor β; MMPs, Matrix Metallo-Proteinases.

Healing or replacing injured tissues is the result of millennia of evolution that has allowed the refinement of increasingly sophisticated processes fundamental to cope with the onslaught of all the biological, physical and chemical challenges that dot our everyday life. Different cell types and a panoply of GFs, cytokines and active metabolites are dynamically coordinated in wound healing. Different sequential steps have been defined to outline such a complex process i.e., hemostasis, inflammation, proliferation, and remodeling/maturation (Figure 1). Platelets are implicated in all these phases, from the early moments, where they are the most abundant cell type present, to the late steps (Wilkinson and Hardman, 2020). Upon tissue injury, platelets rapidly form a fibrin clot that stops bleeding, provides a provisional scaffold for inflammatory cells, and harbors a reservoir of cytokines, chemokines, and GFs that drive the early events of repair, among which the recruitment of neutrophils as the first line of defense against microorganisms. Indeed, within 12–24 h from injury, neutrophils represent ∼50% of all the cells in the wound while after 3–5 days macrophages predominate (Rodrigues et al., 2019). Neutrophils and platelets cooperate to orchestrate the resolution of inflammation by releasing pro-resolving mediators and by polarizing macrophages towards a repair phenotype (Uchiyama et al., 2021). Moreover, platelets release a large array of growth and angiogenic factors, thus playing a role in the proliferative phase. Neovascularization is a pivotal process to meet the high metabolic demands of the healing tissue and is finely regulated by the balanced release of pro- and anti-angiogenic factors. In addition to sprouting angiogenesis induced by the release of VEGF, Hepatocyte Growth Factor (HGF) and Fibroblast Growth Factor (FGF), platelets also stimulate the recruitment of CD34+ bone marrow derived endothelial progenitors through the secretion of Stromal cell-Derived Factor (SDF)-1α (Ho-Tin-Noé et al., 2011). The arsenal of GFs stored in platelet’s granules can aid the proliferation of many cellular protagonists in healing, i.e., keratinocytes in case of wounds or bone cells in case of fractures. Furthermore, platelet-released Transforming Growth Factor (TGF) ẞs and Platelet-Derived Growth Factor (PDGF) act on fibroblasts so that the initial provisional fibrin scaffold is replaced with a granulation tissue rich in immature collagens, fibronectin and proteoglycans (Eisinger et al., 2018). Last, platelets aid the remodeling of the extracellular matrix secreting Matrix Metallo-Proteinases (MMPs) and releasing the hydrolases stored in their lysosomes. Our increasing understanding of the role of platelet in wound healing and tissue regeneration resulted in the development of autologous Platelet-Rich Plasma (PRP) gels, which are largely utilized in a number of clinical settings, from healing of skin wounds and diabetic ulcers to regeneration of tendons and ligaments, from eye lesions to bone loss (Arora and Arora, 2021).

## Platelets in Space

Life on this planet developed and evolved under a static gravity of 9.81 m/s^2^ and surrounded by the atmosphere, a perfect protective shield from radiations. Moving away from Earth, gravity and atmosphere disappear. Therefore, in long-duration space missions, radiations and microgravity represent primary hazards to astronaut’s health (Garrett-Bakelman et al., 2019). Moreover, confinement, isolation, sleep disturbances, alteration of circadian rhythms and possible conflicts among the members of the crew create a variety of potentially stressful demands, known to contribute to a number of diseases (Cohen et al., 2007). We here discussed available evidence on the role of microgravity, radiations and stress on platelets.

### Platelets and Microgravity

Cellular, animal, and human studies performed in simulated or real microgravity demonstrated influences of weightlessness and gravity on haemostasis and, in particular, on platelets, even if available data depict a contrasting picture probably due to the differences in unloading conditions. Briefly, it should be recalled that experiments in real microgravity can be performed only in orbit, i.e., onboard space stations or rockets and capsules, while short duration of reduced gravity can be achieved by parabolic flights (Pletser, 2020). Ground-based analogues for human spaceflight, such as immersion and head down tilt bed rest, are available, but present some limits (Pandiarajan and Hargens, 2020). For rodents, hindlimb unloading is widely used to simulate microgravity (Morey-Holton and Globus, 2002). For studies at the cellular level, several devices, i.e., clinostats, among which the random positioning machine (RPM), and the rotating wall vessel (RWV), are commonly utilized and simulate some aspects of microgravity (for detailed description please see (Maier et al., 2015; Riwaldt et al., 2021) and Table 1.

**TABLE 1:** Summary of the studies on platelets in microgravity using different experimental models.

Model	Method	Type of microgravity	Time of exposure	Effects
Humans	Spaceflight	Real, different g forces experienced	From days to months	Auñón-Chancellor et al. (2020); Limper et al. (2021); Garrett-Bakelman et al. (2019)
	Bed rest	Simulated	From days to months	Brzhozovskiy et al. (2019); Venemans-Jellema et al. (2014)
Animals	Parabolic flight	Real, different g forces experienced	Seconds	Fuse et al. (2002)
	Hindlimb	Simulated	From minutes to days	Dai et al. (2009)
	Spaceflight	Real, different g forces experienced	Days	Davidson et al. (1999)
	RWV	Simulated	From minutes to days	Cialdai et al. (2020)
Cells	Spaceflight	Real, different g forces experienced	Days	Davis et al. (1996); Plett et al.,2001; Plett et al.,2004; Akiyama et al. (1999)
	Parabolic flight	Real, different g forces experienced	Seconds	Schmitt et al. (1993)
	RWV	Simulated	From minutes to days	Li et al. (2010)

The type of microgravity, the duration of microgravity exposure and the results obtained are reported.

#### Platelet, Microgravity, and Hemostasis

Turning to the effects of gravity on platelets, parabolic flight induces thrombocytopenia in mice (Fuse et al., 2002). Two *in vitro* experiment in real microgravity during the missions STS-63 (Discovery) and STS-69 (Endeavour) showed a reduction of the proliferation of CD34+ bone marrow progenitors compared to ground controls (Davis et al., 1996). Consistently, in simulated microgravity, Plett at al. showed inhibition of migration, delay in cell cycle progression with consequent growth retardation and impaired differentiation patterns of hematopoietic progenitors (Plett et al., 2001; Plett et al., 2004), which might account for alterations in platelet counts.

Also platelet functions were investigated in microgravity. Parabolic flight does not inhibit platelet activation. Indeed, Ca^2+^-calmodulin-mediated events and Protein kinase C (PKC) -dependent pathways are maintained. Moreover, no significant modifications in shape changes, phosphorylation patterns or degranulation of platelets were detected after parabolic flight (Schmitt et al., 1993). The limitations of this study are the low level of microgravity reached with parabolic flight (10^−2^ g) and the short time of microgravity experienced (5 or 10 min of cumulative microgravity), which might explain the discrepancy between this and other studies highlighting platelets’ dysfunction in microgravity.

Simulated microgravity decreases platelet adhesion to vWF by downregulating GPIbα on platelet surface (Dai et al., 2009). The interaction of GPIbα with vWF induces different intracellular changes, among which the increase of cytoplasmic Ca^2+^ level. Li et al. demonstrated that different gravity conditions induce a modification of intracellular Ca^2+^ concentration suggesting that platelet intracellular Ca^2+^ plays a key role in the alterations of platelet functions in different gravity conditions (Li et al., 2010). Since GPIbα associates with filamin A, which directly binds the actin cytoskeleton, its downregulation contributes to cytoskeletal disorganization in simulated microgravity. Importantly, cytoskeleton modifications impair platelet functions, reduce platelet release reaction and severely impair platelet aggregation after induction with rystocetin or collagen (Dai et al., 2009).

**FIGURE 2:**
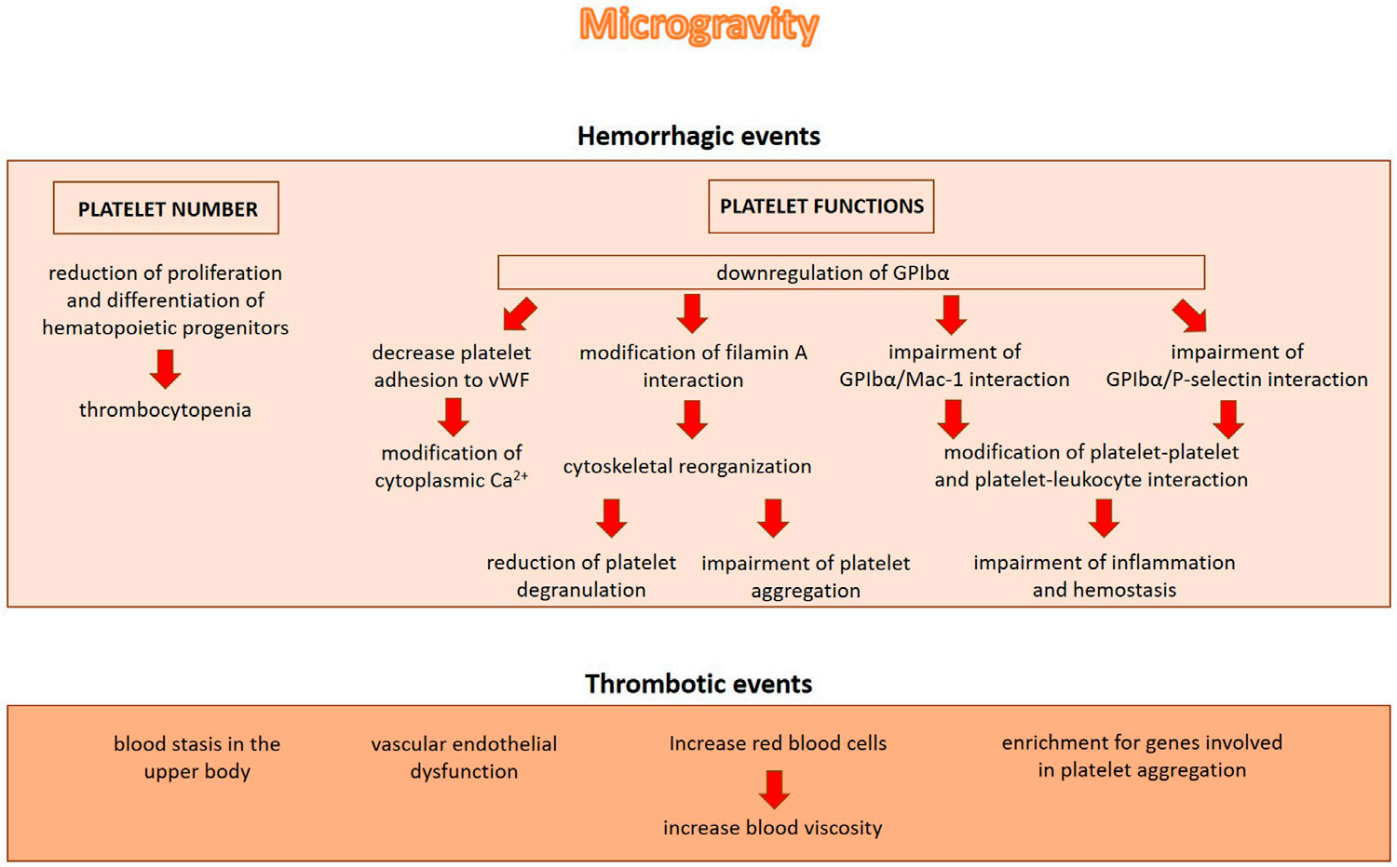
The role of microgravity in inducing hemorrhagic or thrombotic events. Microgravity induces hemorrhagic events by decreasing platelet number and impairing platelet functions. On the other hand, microgravity induces blood stasis in the upper body, vascular endothelial dysfunction and alterations in blood volume and blood viscosity, all events which might contribute to the increase of thrombotic incidents.

In addition to binding vWF, GPIbα also binds the integrin Mac-1 which is expressed on leukocytes. Notably, heterotypic cell—cell interactions between leukocytes and platelets enhance pro-inflammatory and pro-thrombotic events (Wang et al., 2017). GPIbα also binds P-selectin, thus promoting thrombus propagation independently of vVW (Prakash et al., 2017). Therefore, GPIbα downregulation in microgravity suggests an impairment of platelets mediated events in inflammation and hemostasis (Figure 2).

Interesting data were obtained by analysing astronaut’s medical records. A recent study reports the proteomic analysis of blood plasma samples obtained from cosmonauts before and after missions on the international space station (ISS) as well as from volunteers before and after 21 days of bed rest and dry immersion. The study revealed nine common proteins significantly regulated by gravity. Most of these proteins among which SERPIN1, SERPIN3, SERPINC1, SERPING1, A2M, are involved in platelet degranulation being mainly released from α granules (Brzhozovskiy et al., 2019).

Despite the evidence demonstrating that microgravity reduces platelet number and activity, not only hemorrhagic but also thrombotic events have been shown after microgravity exposure. This is not completely surprising given the protective effects exerted by platelets on the endothelium (Nachman and Rafii, 2008). In 2019 the first known blood clot was treated in space. After 2 months onboard the ISS, during an ultrasound examination, an obstructive internal jugular venous thrombosis was revealed in an astronaut (Auñón-Chancellor et al., 2020). The increase of thrombotic incidents may be explained with several factors among which blood stasis in the upper body, vascular endothelial dysfunction and alterations in blood volume and blood viscosity that are documented in real microgravity (Figure 2). When entering weightlessness, the astronauts experience an initial increase in central blood volume, followed by intravascular volume contraction due to reduced thirst and increased urine output. Also the increased levels of fibrinogen *ẞ* chain and the elevated red blood cell counts, both documented in astronauts after long-term spaceflight, might play a role in the formation of thrombi (Limper et al., 2021).

In the NASA Twins Study, the Gene Ontology enrichment analysis revealed an enrichment for genes involved in platelet aggregation and for genes involved in the response to PDGF in the astronaut subjected to one year-long mission compared with his twin on Earth (Garrett-Bakelman et al., 2019). The head-down bed-rest experiments documented reduced deformability and increased aggregation of red blood cell and decreased platelet activation (Venemans-Jellema et al., 2014).

#### Platelets, Microgravity, and Wound Healing

**FIGURE 3:**
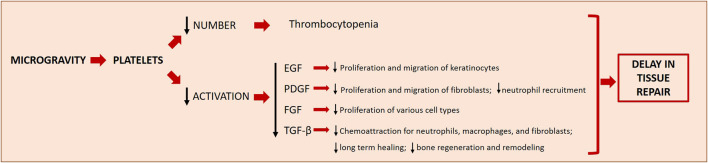
Microgravity effects on platelets and the downstream effects on wound healing. Microgravity induces thrombocytopenia and decreases the activation of platelets, two phenomena which concur to delay tissue repair in space. The decreased production/release of different growth factors (EGF, PDGF, FGF and TGF-β) has downstream effects on other important players of the different steps of wound healing. EGF, Epidermal Growth Factor; PDGF, Platelet-Derived Growth Factor; FGF, Fibroblast Growth Factor; TGF-β, Transforming Growth Factor β.

Wound healing was studied in microgravity using rats subcutaneously implanted with polyvinyl acetal sponge disks releasing PDGF-BB and basic FGF. The capacity to form granulation tissue on the sponge disks was studied during 10 days on the orbiting space shuttle Endeavour. The authors demonstrated a blunted response to the GFs, regarding the cellularity and collagen deposition, in the flight sponges compared to the ground controls. These data suggest that microgravity affects tissue responsiveness retarding the capacity of wound to heal (Davidson et al., 1999). Accordingly, during space flight the response of wounds to PDGF was lower than in control wounds on the ground (Davidson et al., 1999). Similar results have been obtained by an “*in vivo*” model of wound healing based on the use of leeches (Hirudo) exposed to modelled microgravity by RPM, showing a delay in healing capacity probably related to a decrease in collagen fibre density (Cialdai et al., 2020). As mentioned above, wound healing is a complex process orchestrated by the coordinated intervention of many different cell types and by a myriad of different molecules. The initial steps are driven by platelets. Therefore, microgravity-associated thrombocytopenia is itself a critical issue in wound healing. Moreover, microgravity impairs platelet adhesion to the injured tissue, thus reducing the efficiency of clot formation, and blunts the release of growth and angiogenic factors, thus retarding the activation of cell proliferation and migration (Farahani and DiPietro, 2008) (Figure 3). Indeed, platelet-released GFs, such as TGF-*ẞ*, PDGF, and Epidermal Growth Factor (EGF), modulate the healing process. PDGF is mitogenic and motogenic for fibroblasts and stimulates the recruitment of neutrophils (Werner and Grose, 2003). Large amounts of TGF-ẞ are released from platelets immediately after wounding (Werner and Grose, 2003) and this initial kick-start of active TGF-ẞ serves as a chemoattractant for neutrophils, macrophages, and fibroblasts. EGF stimulates keratinocyte migration, fibroblast function and the formation of granulation tissue (Hardwicke et al., 2008). The impaired release of GFs is aggravated by the evidence that microgravity interferes with receptor binding and signal transduction. It is reported that microgravity inhibits EGF-induced signal transduction independently from the redistribution of EGF receptor in the plasma membrane of epidermal cells (Rijken et al., 1993). Also the expression of PDGF receptors and of the various isoforms of TGF-ẞ is downregulated in simulated microgravity (Akiyama et al., 1999; Farahani and DiPietro, 2008). As for TGF-ẞ, since microgravity generates a low shear stress environment, it is likely that altered biomechanical properties decrease TGF-ẞ synthesis. Moreover, flight-induced psychological stress might play a role, because of the increase of glucocorticoids, which are known to inhibit TGF-ẞ transcription (Farahani and DiPietro, 2008).

Furthermore, because fibrin structure determines healing outcomes, it is noteworthy that microgravity affects the branches and porosity of fibrin matrices resulting in the formation of more homogeneous fibrin gels than on ground (Nunes et al., 1995; Roedersheimer et al., 1997). It is feasible that the reduced platelet content in the fibrin plug due to microgravity diminishes the contractile force and facilitates the lysis of the fibrin clot. Platelets are just one of the players in wound healing, but it is clear that their reduced number together with their functional impairment contribute to delay different steps of the process.

### Platelet and Radiations

During space travels, beyond the Earth’s protective magnetosphere, astronauts experience acute and chronic exposure to ionizing radiations, in particular those generated by solar particle event (SPE) (Hu et al., 2009). A SPE is an intense release of ionizing radiation, specifically low energy protons, which occurs in specific regions of the Sun. These radiations represent a serious risk for astronauts mostly during extravehicular activities (Kerr, 2013; Zeitlin et al., 2013). Astronauts experience SPE doses ranging from 0 to 50 cGy/h, and the largest expected tissue dose is ∼2 Gy (Townsend, 2005; Hu et al., 2009). Both *in vivo* and *in vitro* experiments highlight that blood cells are particularly sensitive to ionizing radiation (Dainiak, 2002) and platelets show a gradual decline in number over time (Maks et al., 2011; Romero-Weaver et al., 2013) with a marked drop around day 10 of spaceflight. Platelets number declines more gradually than other circulating cells after irradiation (Maks et al., 2011).

Several studies have reported that the hematopoietic system is particularly sensitive to radiation damage, resulting also in thrombocytopenia (Dainiak, 2002; Stasi, 2012). This seems to be mediated directly, by the direct damage to hematopoietic cells, and indirectly, through the radiation-mediated alteration of endothelial cell function (Wen et al., 2016; Chen et al., 2017). Indeed, the vascular niche is the site of terminal maturation of megakaryocytes and thrombopoiesis (Niswander et al., 2014), where endothelial cells promote the differentiation, maturation and localization of megakaryocytes through the expression of numerous autocrine and paracrine factors (Gaugler et al., 1998; Mazo et al., 2002; Himburg et al., 2016) among which VEGF, whose production was demonstrated to be sensitive to radiation (Chen et al., 2017).

For what regards animal models, Romero-Weaver and colleagues exposed mice to SPE-like proton radiation and/or microgravity simulated by hindlimb unloading and analyzed the number of different blood cells among which platelets (Romero-Weaver et al., 2014). They found that platelet counts were decreased significantly in a dose dependent manner by proton radiation, but not significantly affected by hindlimb unloading treatment alone. Exposure to radiation alone caused a significant decrease in platelet number with a drop on day 10 post-irradiation. This reduction in platelet numbers might increase the risk of hemorrhagic events and, eventually, delay healing (Dai et al., 2009).

Of note, differences determined by gender were found in platelets response to proton radiation by Billings and colleagues. They show that non-irradiated female mice have 13% less platelets than their male counterparts and recover slower than males post irradiation. Both males and females display platelet decrease at day 4 post irradiation with a drop at days 11–12, followed by a consistent rebound in males and a slower in females (Billings et al., 2014). The opposite gender trend was reported in humans (Liu et al., 2021) analyzing the effects of low cumulative doses of radiations on medical workers. Males have lower platelet counts than females, but they both display a first increase post irradiation and then a decrease in the number of platelets, in a dose-dependent manner. It is important to note that platelet aggregation and release reaction were not altered by the irradiation (Rock et al., 1988).

### Platelet and Psychological Stress

Psychological and social issues affect astronauts proportionally to the duration of the space mission (Oluwafemi et al., 2021). Not only isolation, confinement, separation from families and interpersonal tensions but also sleep disorders generate stress. Numerous studies have shown a relation between psychological stress and somatic disorders (Kendler et al., 1999; Leonard, 2006; Anisman, 2009; Haroon et al., 2012). First of all, stress activates the hypothalamic-pituitary–adrenal (HPA) axis, resulting in increased levels of glucocorticoids which impair wound healing (Slominski and Zmijewski, 2017). This is due to the fact that glucocorticoids inhibit inflammation, required in the early phases of repair, and retard keratinocyte migration and wound closure (Slominski and Zmijewski, 2017). However, glucocorticoids exert no effects on platelet number and functions (Liverani et al., 2012).

Platelets contain the largest amount of serotonin (5-HT) outside the central nervous system and express serotonin receptors 2A and 3A (5-HT-2A receptor, 5-HT-3A receptor), α-2, β-2 adrenoreceptors, benzodiazepine and the serotonin transporter (SERT) (Camacho and Dimsdale, 2000). Their activation might occur, in addition to the canonical pathways, by various lifestyle factors such as physical and mental stress (Camacho and Dimsdale, 2000; El-Sayed, 2002; Jurk and Kehrel, 2005). The HPA axis also activates the sympathetic nervous system (SNS) and the serotonergic system, thus activating platelets. Increased platelet activity is reported in emotional stress through increased serotonin binding to 5-HT-2 receptors on platelets (Markovitz and Matthews, 1991; Garvey et al., 1995) or to increased platelet 5-HT reuptake, as described in patients with anxiety or depression (Pecknold et al., 1988). In general, it seems that stress does not importantly affect platelet function and, therefore, stress associated delay of wound healing is mainly due to effects on all the other cell types involved in the process (Gouin and Kiecolt-Glaser, 2011).

## Platelet Rich Plasma: The Future of Wound Healing in Space?

With the increase of manned missions in space, the chances of injury due to traumatic events or unexpected emergency surgery will increase. Wound healing might represent one of the major problems onboard, and this raises the need to promote studies to define adequate countermeasures. As mentioned above, a great amount of data demonstrate the improvement of tissue repair and wound healing upon PRP application (Arora and Arora, 2021). These outcomes together with the feasibility to obtain PRP (once prepared from a few milliliters of autologous blood, it can be stored frozen for many months onboard) indicate that PRP might become a new challenge in the field of tissue healing also in space.

**FIGURE 4:**
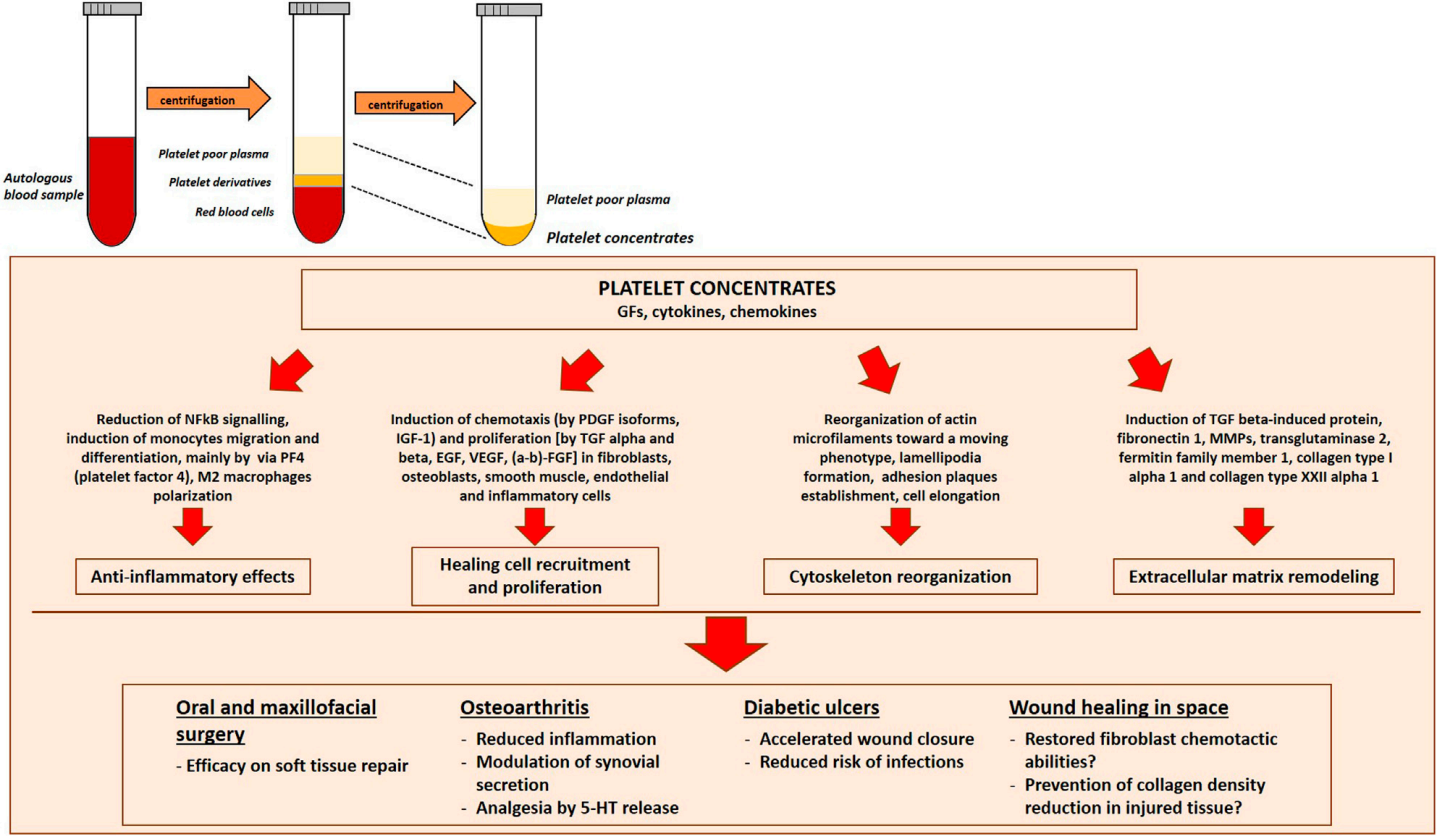
Platelet Rich Plasma (PRP) preparation and efficacy in different clinical conditions. PRP is prepared by centrifugation of peripheral blood to concentrate platelets. Platelet derivatives are promising tools to promote regeneration and healing for future space travel. Indeed, they have yielded excellent results in different medical fields, from oral and maxillofacial surgical procedures, to the treatment of symptomatic osteoarthritis patients and diabetic ulcers. NFκB, Nuclear Factor kappa B; TGF-β, Transforming Growth Factor β MMPs, Matrix Metallo-Proteinases, 5-HT, serotonin.

The rationale of using PRP relies on the evidence that, upon degranulation, platelets release GFs, thereby accelerating the recruitment of the cells implicated in the healing process. Indeed, PRP application yielded excellent results in oral and maxillofacial surgical procedures for its efficacy on soft tissue repair (Mijiritsky et al., 2021). Moreover, autologous PRP represents a promising adjuvant therapy for the treatment of diabetic foot ulcers, since it ameliorates the healing process and reduces the risk of infections (Shao et al., 2020). In addition, PRP has been proposed as a local supply of cytokines and GFs in different hyaluronan-controlled and placebo-controlled clinical trials (Andia and Maffulli, 2013) in patients with symptomatic osteoarthritis, even if the cellular and molecular mechanisms of PRP effects remain poorly elucidated. Furthermore, PRP shows anti-inflammatory effects likely by interfering with Nuclear Factor kappa B (NFkB) signaling (Andia and Maffulli, 2013), increases cartilage height and reduces the loss of cartilage matrix by diminishing chondrocyte apoptosis (Testa et al., 2021). Moreover, PRP ability to polarize macrophages towards a M2 repairing phenotype is of utmost importance to reduce the proinflammatory chronic effects of joint M1 macrophages (Uchiyama et al., 2021) (Figure 4).

Very few studies are published concerning the application of platelet derivatives in different gravity conditions. PRP treatment can partially counteract microgravity-induced alterations in cultured fibroblasts (Cialdai et al., 2020). When applied to a fibroblast cell culture exposed to simulated microgravity in RPM, PRP prevents the formation of 3D aggregates, probably by remodeling the cytoskeleton (Casati et al., 2014) and restores, at least in part, the chemokinetic abilities of fibroblasts largely compromised by gravitational unloading (Cialdai et al., 2020). The regenerative effects of PRP were recently proved also in an “*in vivo*” model of tissue repair in microgravity, based on the use of leeches (Cialdai et al., 2020), considered a good model for the study of tissue repair (Tettamanti et al., 2004) as the wound healing process occurs similarly to vertebrates. To mimic a wound, a surgical lesion (length 10 mm, depth ∼2 mm) was performed on the dorsal skin of each leech before exposing them to simulated microgravity in the RPM. The supply of PRP to the medium prevented both healing delay and alterations in tissue structure, narrowing the surgical wound, enhancing re-epithelization and preventing the decrease in collagen meshwork density of collagen fibers of the peri-lesional connective tissue (Cialdai et al., 2020). The first results are promising, but more studies are necessary to assess PRP usefulness in microgravity.

## Conclusion

Since 1971, astronauts have spent months in space stations orbiting Earth. Future human missions to Mars will require astronauts to live in space more than 2 years. A human outpost is foreseen on the moon to be used as a long-term foothold for lunar exploration and as a gateway for deep space missions. Moreover, if and when Earth is incapable of supporting life, space might be an alternative for human survival. Last, 20 years after the first space tourist, more people are experiencing spaceflight. Therefore, long term permanence and more people in space require to optimize how to face the most likely injury that might occur, i.e., a wound. PRP might represent a safe, relatively easy and efficient tool to accelerate tissue repair in an extreme environment. Only few data are available at the moment to prove its effectiveness also during weightless condition and further studies are needed to test whether long term storage of PRP in space affects the stability and activity of the GFs and cytokines and, thus, PRP regenerative properties.
